# Preparation and Performance of AgNWs/PDMS Film-Based Flexible Strain Sensor

**DOI:** 10.3390/ma16020641

**Published:** 2023-01-09

**Authors:** Xiaoxin Zhu, Yimin Zhou, Cui Ye

**Affiliations:** College of Materials Science and Engineering, Zhejiang University of Technology, Hangzhou 310014, China

**Keywords:** silver nanowires (AgNWs), AgNWs/PDMS film, strain sensor, flexible and wearable devices

## Abstract

Flexible strain sensors are widely used in the fields of personal electronic equipment and health monitoring to promote the rapid development of modern social science and technology. In this paper, silver nanowires (AgNWs) prepared via the polyol reduction method were used to construct a flexible strain sensor. The AgNWs/PDMS film was obtained by transfer printing using AgNWs as a conductive layer and polydimethylsiloxane (PDMS) as a flexible substrate. The morphology of AgNWs was characterized by SEM and TEM. The aspect ratio of the AgNWs was more than 700. The strain sensitivity factor of the sensor was 2.8757, with a good linear relationship between the resistance and the strain. Moreover, the strain sensor showed good response results in human activity monitoring and the LED lamp response test, which provides a new idea for the construction of flexible wearable devices.

## 1. Introduction

With the advent of 5G and the Internet of things era, smart wearable devices are also at a stage of rapid development. Flexible wearable electronic devices with stretchability, high efficiency, and low-cost manufacturing technology, have a wide range of application prospects in information, energy, medical care, national defense, and other fields, such as flexible electronic displays, organic light-emitting diodes (OLEDs) [1], printed radiofrequency identification (RFID), thin-film solar panels, flexible sensors [2], and flexible electronic skin [3]. As for the integrated system of intelligent wearable devices, flexible sensors play a vital role. According to wearable flexible sensors that can be used to detect biological signals, they can be divided into three categories: electrophysiological sensors, chemical sensors, and physical sensors. Electrophysiological sensors are usually used to detect physiological signals such as electroencephalograms (EEGs), electrocardiograms (ECGs), and electromyographs (EMGs) [4]. Chemical sensors are commonly used to detect common chemicals in human body fluids, such as glucose [5], lactate [6], alcohol [7], medicine content in human metabolism process [8], and pH of body fluids [9]. Physical sensors are usually flexible and respond visually to strain [10], temperature [11], humidity [12], sound [13], etc., making the detection of physical signals more convenient and accurate, so as to realize the accurate monitoring based on the normal physiological activity signals of the human body, and achieve the purpose of disease prevention and health monitoring. It is an extremely complex process for intelligent wearable devices to realize accurate environmental perception, which requires different types of sensors to play a synergistic role to complete the detection of physical signals, physiological signals, and chemical signals in the wearable system [14].Compared with flexible chemical sensor and flexible physiological sensors, flexible strain sensors have been widely studied because of their wide application, simple preparation process, and easy-to-capture strain signal. The complex preparation technology and performance of flexible strain sensors directly affect their practicability. Therefore, an effective prerequisite for improving the performance of strain sensors is the preparation technology, which needs to be optimized from the selection of sensor materials and the construction of sensor components.

A silver nanowire (AgNW) is a single crystal structure with an average diameter of 20–100 nm and average length of 20–200 μm, with a similarly high electrical conductivity (6.3 × 10^−7^ S·m^−1^) and thermal conductivity (429 W·m^−1^·K^−1^) to silver [15]. AgNWs are usually prepared via the polyol reduction method, and the aspect ratio can be adjusted in a certain range, while the yield and purity are also improved steadily. When it is coated on polyethylene terephthalate (PET), the visible-light transmittance of the AgNWs/PET film is up to 90%, and an effective conductive channel is crearws with a block resistance of less than 30 Ω/m. As the diameter of AgNWs decreases, the scattering of visible light decreases gradually, and the fog can be reduced to less than 0.5% [16]. The properties of transparent conductive films based on AgNWs are expected to become the most important flexible conductive materials in the field of flexible electronics.

A stretchable conductive film is usually composed of conductive film and flexible substrate; polydimethylsiloxane (PDMS) is the most common flexible substrate material with excellent tensile properties. However, PDMS is extremely hydrophobic, and it is difficult to form thin films on the surface of PDMS [17]. In order to solve this problem, researchers have targeted two approaches: (1) the conductive material is directly spread on the prepared PDMS film, and then another layer of PDMS film is added for packaging; (2) after forming a thin film on a film-prone substrate, the conductive material is combined with PDMS by means of physical transfer. Pegah Hashemi et al. [18] used a large amount of AgNWs suspension on a cured PDMS/thermoplastic polyurethane (TPU) film, and covered with PDMS/TPU to form a sandwich film. The strain sensitivity factor (GF) of the obtained sensor was as high as 8.32. However, the durability and recyclable performance of sensors need to be improved. Fang et al. [19] sprayed carbon nanotubes (CNT) onto a polytetrafluoroethylene (PTFE) film and then covered it with uncured PDMS. After PDMS curing, it was removed and prepared to obtain the PDMS/CNT film, which completed the key step of the sensor construction. However, PTFE film is easily damaged in the process of stripping, which affects its electrical sensing performance. Moreover, a critical requirement for the film sensor is to demonstrate a high level of wear resistance (flexural and tensile), while maintaining its function and reliability under large mechanical deformation [20]. Therefore, there is an urgent need to develop a simple and efficient method for the preparation of two-dimensional (2D) thin-film sensing elements, which can completely preserve its conductive path.

On this basis, this paper mainly discusses the preparation of 2D thin-film strain sensors by physical transfer printing. AgNWs/PET films were prepared on polyethylene terephthalate (PET) substrates by scraping and coating. At the same time, AgNWs/PDMS films with complete morphology and structure were prepared via low-temperature transfer printing using PET as a transfer medium and PDMS as a flexible substrate. Thus, the electrical and mechanical properties of AgNWs/PDMS films were further optimized, and a strain sensor based on 2D AgNWs/PDMS films was prepared. With excellent sensing performance and a wide detection range, the sensor can visualize the strain response by observing the brightness changes of LED lights, which is expected to be applied to the monitoring of human limb movement.

## 2. Materials and Methods

### 2.1. Materials and Apparatus

The experimental materials used in this experiment were silver nitrate (AgNO_3_), ethylene glycol (EG), and copper chloride (CuCl_2_·2H_2_O), purchased from Sinopharmaceutical Chemical Reagent Co., Ltd. (Nanjing, China) Polyvinylpyrrolidone (PVP-55000, average molecular weight: 55,000; PVP-360000, average molecular weight: 360,000) were purchased from Sigma Aldrich Trading Co., Ltd. (St. Louis, MO, USA). Polydimethylsiloxane (PDMS, DOWSIL 184 silicone elastomer base and matched silicone elastomer curing agent) was supplied by Dow Corning (Midland, MI, USA).

As for the experimental instruments, the KQ-500DA ultrasonic cleaner was purchased from Kunshan Ultrasonic Instrument Co., Ltd. (Kunshan, China). The TQ16-WS centrifuge was obtained from Hunan Xiangyi Laboratory Instrument Development Co., Ltd. (Changsha, China). The HZ-1007C computer desktop servo testing machine (Dongguan Lixian Instrument Technology Co., Ltd. Dongguan, China) was applied to detect the stress–strain curve; The Four-Point Probe Resistivity Measurement System (4Probes Technology Co., Ltd. Guangzhou, China) and 2450 SourceMeter (Tektronix Co.,Ltd. Shanghai, China) were used to detect the performance of the film sensor. Moreover, the instruments used for the characterization of the experimental samples in this paper were a field-emission scanning electron microscope (SEM) and transmission electron microscope (TEM) purchased from FEI in the United States, model Nova nano 450 (Hillsboro, OR, USA).

### 2.2. Preparation and Purification of AgNWs

(1) Preparation of AgNWs

AgNWs were synthesized using the polyol method reported by our research group [16]. A slight improvement was made in the synthesis process of silver nanowires with a diameter of about 70 nm. First, CuCl_2_ solution (0.0132 g, 16 mL ethylene glycol) and AgNO_3_ solution (0.9 g, 20 mL ethylene glycol) were prepared using an ultrasonic cleaner in an ice bath. Then, 116 mL of ethylene glycol was measured using a graduated cylinder and placed in a 250 mL three-neck round-bottom flask. Next, 0.406 g PVP-360000 and 0.421 g PVP-K30 were added to the flask, which was placed in a heating jacket at 130 °C to completely dissolve PVP. After PVP was dissolved, the heating temperature was set at 140 °C, and the above-prepared CuCl_2_ solution (3.2 mL) and AgNO_3_ solution (20 mL) were injected into the flask (injection time was controlled within 5 min). After reaction for 50 min, the flask was removed and quenched to room temperature in water.

(2) Purification of AgNWs

AgNWs synthesized via the polyol method contain fewer impurities such as particles and rods; hence, the centrifugal method was used for purification. The specific process was as follows: first, 20 mL of AgNWs were added to 20 mL of deionized water, and separated at 3500 rpm for 5 min in a centrifuge. Then, the supernatant was removed (removing the residual organic liquid and part of the particle short rod), and 5 mL of ethanol was added into the sediment. After mixing thoroughly, it was directly used as the AgNWs coating solution. The purification step was executed, and most of the PVP was wiped off. The residual PVP on the surface of the AgNWs is beneficial for two reasons. The residual PVP coating on the surface of AgNWs acts as a protective layer, which can prolong the storage time of AgNWs and avoid their rapid oxidation [16]. Additionally, PVP contains the useful functional groups for bonding, which can ensure the integrity of film formation during the AgNWs coating process [21].

### 2.3. Preparation of Strain Sensors Based on AgNWs/PDMS Films

Firstly, AgNWs/PET films were prepared by scraping and coating, and then AgNWs/PDMS films were prepared using the low-temperature transfer method. The specific operation involved first cutting the PET film into a size of 10 cm × 12 cm. After ultrasonic cleaning, it was cleaned with ultraviolet ozone for 40 min, and then placed on the automatic coating machine. Next, 1.5 mL of the silver nanowire dispersion solution was taken for use, and the coating speed was set to 60 mm·s^−1^. After coating, the AgNWs/PET film was dried in an oven at 60 °C for 5 min. Then, PDMS (5:1/10:1/20:1/40:1) was prepared by changing the mass ratio of the main agent and the curing agent (DOWSIL 184 silicone elastomer base and silicone elastomer curing agent). After stirring, the liquid PDMS precursor was covered on the AgNWs/PET film, before curing in oven at 80 °C for 3 h. The resulting PET/AgNWs/PDMS film was placed in liquid nitrogen for 2 min, and AgNWs were transferred from PET to PDMS through the glass transition behavior at low temperature to obtain the AgNWs/PDMS film [19].

### 2.4. Strain Sensing of the AgNWs/PDMS Films

The strain test involved in our work was mainly performed using a HZ-1007C computer desktop servo testing machine combined with a 2450 SourceMeter. The samples were prepared by cutting the AgNWs/PDMS film into 2 cm × 4 cm samples, and pasting the copper strips on both ends of the film with the help of conductive silver paste to form a conductive path. At a stretching speed of 30 mm·min^−1^, the strain test was carried out, and a change in resistance was recorded using the software.

## 3. Results and Discussion

### 3.1. Morphology Characterization of the of AgNWs/PDMS

**Figure 1:**
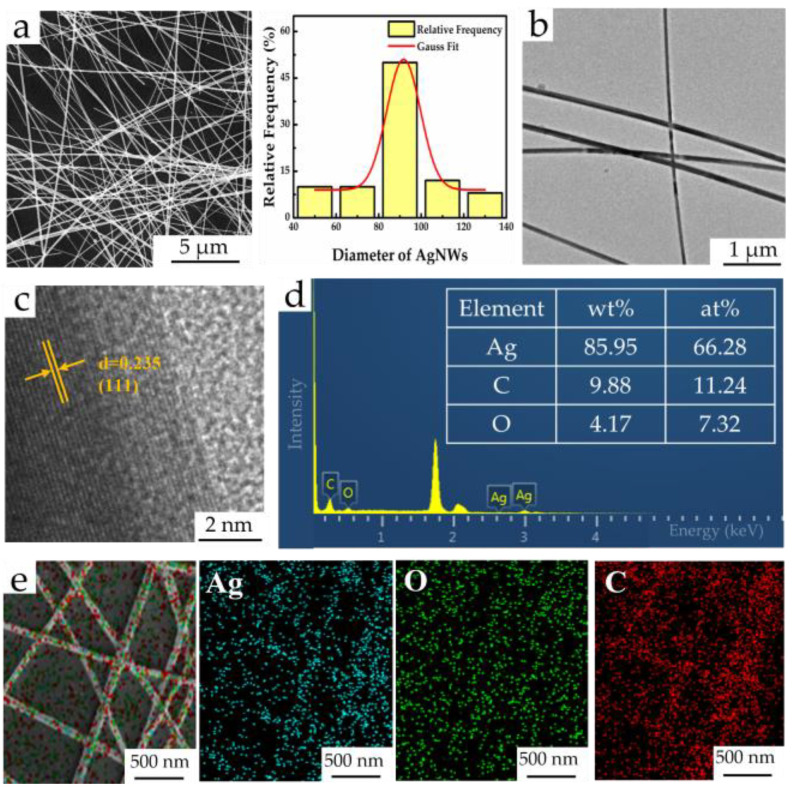
(**a**) SEM image and Gaussian statistical distribution of diameter; (**b**) TEM image; (**c**) HRTEM image; (**d**) EDS composition chart; (**e**) element distribution maps of AgNWs.

Figure 1a,b show the SEM and TEM images of the AgNWs; the prepared AgNWs had a relatively uniform diameter and fewer byproduct silver nanoparticles. The Gaussian statistical distribution also shows that the diameter of AgNWs was around 90 nm. Figure 1c shows the HRTEM image of AgNWs, and the measured crystal plane spacing was 0.235 nm, corresponding to the (111) crystal plane [22]. Figure 1d,e are the corresponding EDS composition chart and element distribution maps of AgNWs. The results show that the product was mainly composed of Ag, and there were no other impurities except for C and O caused by PVP residue on the surface of AgNWs. Figure S1a shows the AgNWs/PDMS film after frozen stripping in liquid nitrogen. As can be seen, the flexible film prepared using this spalling method was relatively complete, and there was almost no residue on the PET after stripping (Figure S1b). The AgNWs/PDMS film obtained using the low-temperature transfer method had good electrical conductivity (about 5.36 Ω of the initial resistance), laying a foundation for the subsequent construction of the 2D AgNWs/PDMS film strain sensor.

### 3.2. Optimization for the AgNWs/PDMS Films

**Figure 2:**
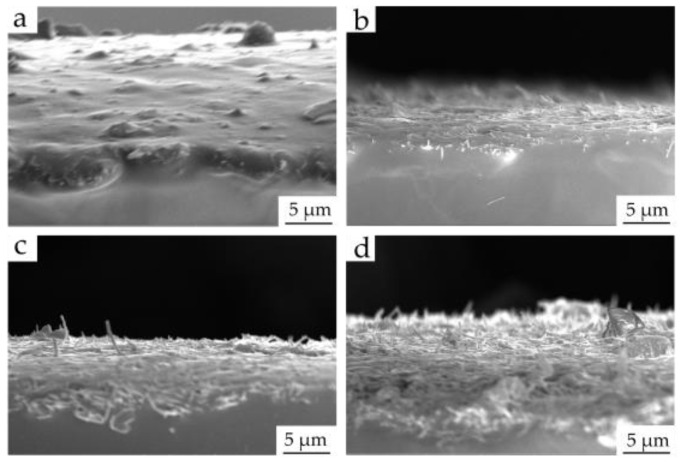
SEM images of AgNWs/PDMS films with different mass ratios of PDMS main agent and curing agent: (**a**) 40:1; (**b**) 20:1; (**c**) 10:1; (**d**) 5:1.

In order to further study the effects with different mass ratios of main agent and curing agent (DOWSIL 184 silicone elastomer base and silicone elastomer curing agent) on the electrical conductivity, we used scanning electron microscopy (SEM) to characterize the morphology of four kinds of films. As can be seen in Figure 2, when the mass ratio of main agent to curing agent was 40:1 and 20:1, the AgNWs on the surface was almost completely covered during the curing process of PDMS. As a result, AgNWs could not form a good conductive network on the surface. In contrast, although the AgNWs on the films with mass ratios of 10:1 and 5:1 were partially covered by PDMS, bare AgNWs could still be obviously observed on the surface; therefore, these two ratios had an advantage in conducting electricity.

**Figure 3:**
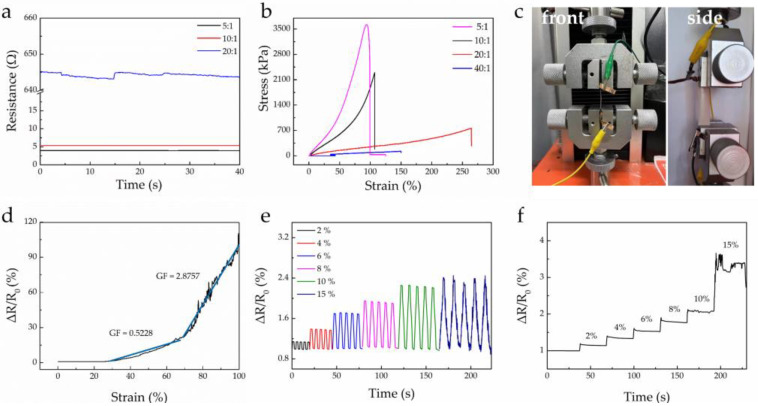
(**a**) Initial resistance values of AgNWs/PDMS films, and (**b**) stress–strain curves of AgNWs/PDMS films with different mass ratios of main agent and curing agent. (**c**) Digital photograph of AgNWs/PDMS film during the stretching process. (**d**) Tensile resistance variation. (**e**) The cyclic response and (**f**) the step response of AgNWs/PDMS thin-film sensor at 2%, 4%, 6%, 8%, 10%, and 15% strain.

To verify whether the mass ratio of main agent to curing agent affected the conductivity of AgNWs/PDMS films, AgNWs/PDMS films were prepared with mass ratios of 40:1, 20:1, 10:1, and 5:1. The initial resistance tests were carried out on the films using a Four-Point Probe Resistivity Measurement System. After the thickness of the film was entered into the program, the dual electrical measurement software started to work automatically, in which the voltage value was collected in real time. The resistance value was calculated according to the built-in formula of the software. As shown in Figure 3a, when the mass ratio of main agent to curing agent is 5:1, the initial resistance value of the film was only 3.98 Ω. When the mass ratio was 10:1, the initial resistance value of the film was about 5.36 Ω, and the initial resistance values are similar under the two mass ratios. When the mass ratio increased to 20:1, the initial resistance value of the film was about 645 Ω. Compared to the initial material, this represented an increase of nearly two orders of magnitude. It is worth noting that the initial resistance of AgNWs/PDMS films exceeded the instrument’s maximum range, and no data are shown here, as the ratio of primary agent continued to increase to 40:1. According to the results in Figure 2, a higher proportion of curing agent led to more AgNWs being exposed on the surface of AgNWs/PDMS film, thus improving the conductivity [23,24].

As a flexible substrate of flexible strain sensor, the tensile property of PDMS largely determines the tensile properties of the strain sensor [24]. Therefore, the tensile properties of the four films with different mass ratios were tested and analyzed. Figure 3b shows the stress–strain curves of AgNWs/PDMS films at four scales, and Figure 3c displays a photograph of the stretched film generated by the HZ-1007C computer desktop servo testing machine. As can be seen, AgNWs/PDMS films had good tensile properties and could withstand about 100% strain. Among them, the AgNWs/PDMS film with the mass ratio of main agent to curing agent of 5:1 could withstand the maximum stress. When the stress reached 3609 kPa, the corresponding strain was 93%. The AgNWs/PDMS film with a mass ratio of 20:1 could withstand the maximum strain, reaching up to 264%. If the elastic modulus of a film is too large, a change in resistance is not obvious when the body generates strain. This phenomenon can greatly affect the sensing performance of strain sensors based on thin films. Considering the practical application, the stress–strain behavior of the skin was about 0.1–2 MPa [25,26], and the stress could reach 2298 kPa with a mass ratio of 10:1, indicating that the film can detect the signals on the surface of the skin before the breaking limit. The above results proved that the proposed senor can be used to study the stress–strain behavior of human skin, thus ensuring its reliability in real application. Therefore, the film with a mass ratio of main agent to hardener of 10:1 was selected for the subsequent construction, performance, and application research of strain sensor.

The sensing mechanisms of thin-film strain sensors can basically be divided into crack type [27], fold type [28,29], and pattern type [30,31]. In order to explore the tensile strain sensing mechanism of AgNWs/PDMS films, we stretched the films under the optimal mass ratio (10:1) and characterized the surface morphology. As shown in Figure S2, obvious folds appeared on the surface of the film, and the results basically confirmed that the sensing mechanism of the AgNWs/PDMS film was fold type. In addition, AgNWs bent rather than broke on the surface during stretching, indicating that the fold did not unduly affect the conductivity of the entire film. Therefore, strain sensors based on AgNWs/PDMS films are expected to show better sensing performance.

### 3.3. Performance of the Strain Sensors

After exploring the electrical and mechanical properties of AgNWs/PDMS films, we continue to investigate the sensing properties of strain sensors based on the films. The gauge factor (GF value) is a very important parameter for flexible strain sensors. The calculation formula of the GF value is as follows:

(1) $$GF=\frac{\delta \left(\Delta R/R_{0}\right)}{\delta \varepsilon },$$

where $R_{0}$ is the initial resistance before applying external force, ΔR is the relative change value of resistance before and after applying external force, δε is the relative change value of strain after applying pressure, and $\Delta R/R_{0}$ is the rate of relative resistance change calculated by the relative change value of resistance and the initial resistance. The device used for the relative resistance measurement was an HZ-1007C computer desktop servo testing machine. When the corresponding external force was applied to the film, the changes in resistivity could be obtained using the 2450 SourceMeter under different stresses. At a stretching speed of 30 mm·min^−1^, a tensile test of the strain sensor based on the AgNWs/PDMS thin film was carried out. As shown in Figure 3d, the GF was 0.5228 between 28% and 67% strain, but 2.8757 when the strain exceeded 67%. The fluctuation of the curve below 28% was not obvious; hence, it was not deemed necessary to further show the linear fitting. In comparison, the GF value of TPU and ABS materials used in a similar study was 2–3 in the strain range of 0–0.2 [32].The results indicate that the AgNWs/PDMS film is sensitive to the detection of resistance changes under strain (converting mechanical signals into electrical signals during the stretching process).

In addition, we tested the resistance of the sensor under different strains. Figure 3e shows the resistance of the sensor under the tensile cyclic strains of 2%, 4%, 6%, 8%, 10%, and 15%. The variation of resistance under different strains was quite different, indicating that the strain sensor could respond to the corresponding strain more accurately. Under the same strain, the resistance changes were stable when repeated for five cycles. Figure S3 shows the result of the stretching–releasing tests of the strain sensor. The relative resistance changes of the sensor remained stable, demonstrating the good stability of our strain sensor. It is worth saying that the shape and height of each peak were similar, indicating the durability [33]. Figure 3f shows the continuous step of the resistance under a continuous strain of 2%, 4%, 6%, 8%, 10%, and 15%, indicating that the strain sensor had the ability to continuously respond to different strains. Moreover, as can be seen in Figure S4, the shape and height of each peak were similar, indicating the durability. The lengths of the film before and after stretching were 4.1 cm and 4.15 cm, respectively, and no significant deformation occurred in the film. The specific calculation process of the recovery rate is as follows:

(2) $$Recovery\;Rate=\frac{L_{p}-L_{0}}{L_{0}}=\frac{4.15-4.1}{4.1}=1.22\%,$$

where *L*_0_ and *L_P_* are the lengths of film before and after stretching. Compared with other published work (Table S1), the prepared AgNWs/PDMS film showed a good recovery rate.

### 3.4. Application Study of Strain Sensor

The strain sensor monitors different strains not only through changes in resistance, but also through the brightness of the LED. We connected the LED with the word “ZJUT” to the circuit and recorded the change in resistance and brightness of the LED during the stretching of the sensor. As shown in Figure S5, with the increase in strain value (0%, 2%, 4%, 6%, 8%, 10%, and 15%), the change in resistance gradually increased. At the same time, the brightness of the LED gradually decreased from extremely bright to slightly bright, which directly illustrates how the strain sensor converted mechanical signals into electrical signals. In addition, in order to further explore the detection capability of the strain sensor, we installed it on a naturally deflated balloon and applied the voltage of 1 V. Then, we inflated the balloon and recorded the resistance changes during the inflation process. As can be seen in Figure S6, as the volume of balloon increased, the strain perceived by the sensor also increased, along with the resistance. The results show that the sensor could provide a direct resistance response to some natural strains occurring in daily life.

**Figure 4:**
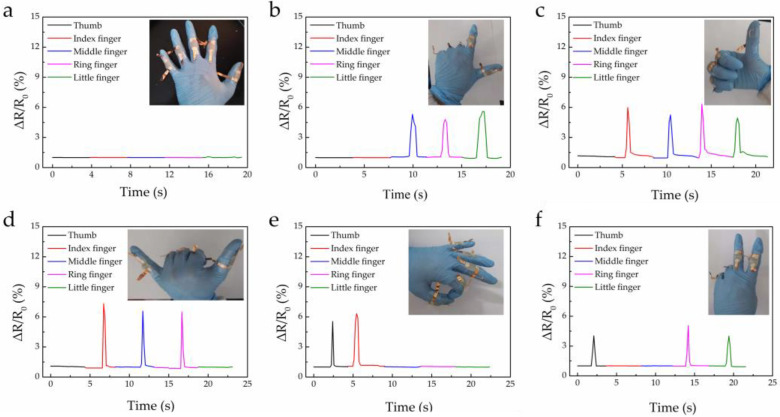
Resistance response to different gestures of human body for strain sensor.

In order to explore the potential application of the strain sensor in the monitoring of human movement, we affixed it to the knuckle of the hand under the condition of applying a constant voltage of 1 V, and we made different gestures to observe the change in resistance signal (Figure 4). When the five fingers were naturally extended, the sensor was in the initial state and the resistance hardly changed (Figure 4a). When making different gestures, the states of the five fingers differed, and the corresponding resistance also changed. As shown in Figure 4e, when the fingers made the “OK” gesture, the thumb and index finger were bent, and the resistance changed obviously. Since the middle, ring, and little fingers were naturally extended, resistance changes we almost zero. Of course, since they were all strains at the finger joints, the rate of resistance change was almost about 5.2. When the fingers made other different gestures (Figure 4b–d,f), bending the finger also changed the corresponding resistance, while extending the finger remained in its original state. According to our previous work [21], we developed a PET/AgNWs/PDMS yarn for monitoring movements in humans, and a stable and repeatable response could be observed, indirectly proving the stability of our work using the AgNWs-based film in the finger gesture test.

## 4. Conclusions

In this work, AgNWs/PDMS films were successfully prepared using the low-temperature transfer method, and the conductivity and tensile properties of AgNWs/PDMS films were improved by optimizing the mass ratio of the main agent and curing agent of PDMS. A higher proportion of curing agent led to more AgNWs being exposed on the surface of AgNWs/PDMS film, thus improving the conductivity. When the mass ratio was 10:1, the resistance of the AgNWs/PDMS film was only 5.36 Ω, showing excellent conductivity. The strain sensor based on a thin film and the good tensile properties of PDMS yielded a tensile strain greater than 100%. The GF value of the strain sensor based on the AgNWs/PDMS film could reach 2.8757. In addition, the sensor showed excellent strain response characteristics in LED lamp and balloon inflation/deflation experiments. When applied to human activities, the sensor could show the changes of finger gestures more accurately. This sensor provides a new idea for flexible structures based on strain response and is expected to be applied in the field of flexible wearable sensing.

## Data Availability

Not applicable.

## Supplementary Materials

The following supporting information can be downloaded at: https://www.mdpi.com/article/10.3390/ma16020641/s1. Refs. [34,35,36] are cited in the supplementary materials. Figure S1: (**a**) AgNWs/PDMS film, (**b**) PET after strippingReferences; Figure S2: SEM images of AgNWs/PDMS film stretched at the magnification of 6000× (**a**) and 1000× (**b**); Figure S3: (**a**) The stretching-releasing tests of the strain sensor, (**b**) The stretching-releasing tests of 10 cycles. Figure S4: (**a**) The stress variation of the AgNWs/PDMS film in a typical bending cycle of 0–30% strain, (**b**) The stress variation for 10 cycles, (**c**) Photographs of the stretching process; Figure S5: Brightness change of LED lamp during strain sensor stretching at 0%, 2%, 4%, 6%, 8%, 10%, 15% strain. Figure S6: Resistance changes of strain sensor during balloon inflation; Table S1: Recovery rate of recently reported PDMS strain sensors.
