# Supplementary material for: Preparation and Performance of AgNWs/PDMS Film-Based Flexible Strain Sensor
Source: Materials (Basel). 2023 Jan 9;16(2):641. doi: 10.3390/ma16020641 (PMC9862830; doi:10.3390/ma16020641)
Supplement: Supplementary file 1 [file materials-16-00641-s001.zip › materials-2108362-supplementary.pdf]

materials

Supplementary Materials

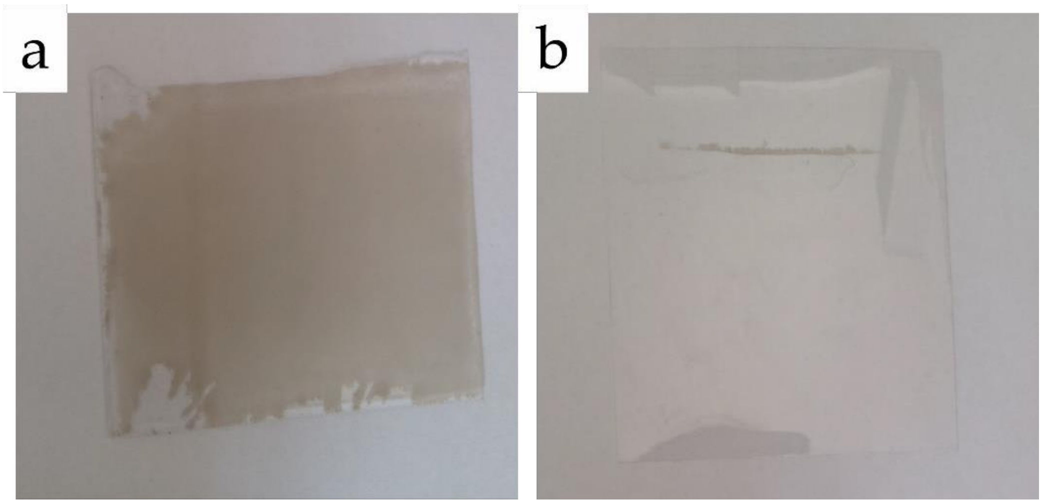

Figure S1. (**a**) AgNWs/PDMS film; (**b**) PET after strippingReferences.

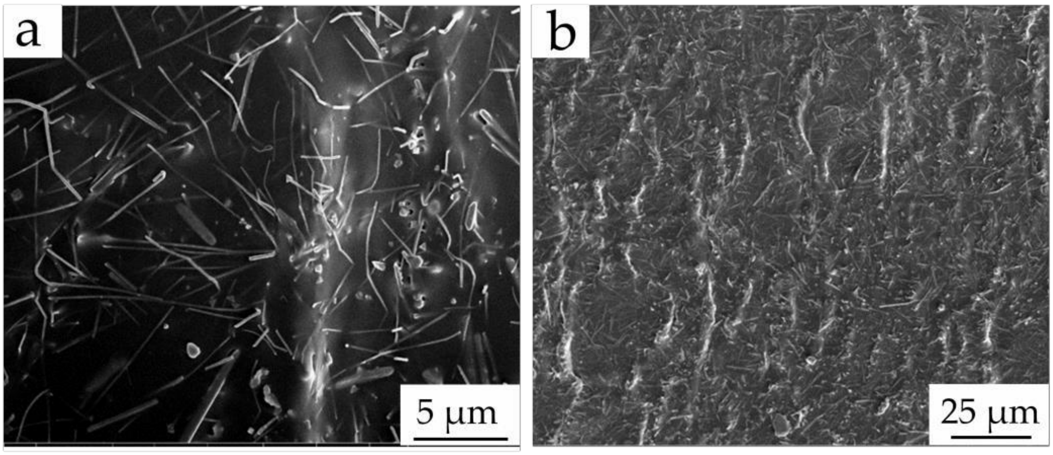

Figure S2. SEM images of AgNWs/PDMS film stretched at the magnification of 6000× (**a**) and 1000× (**b**).

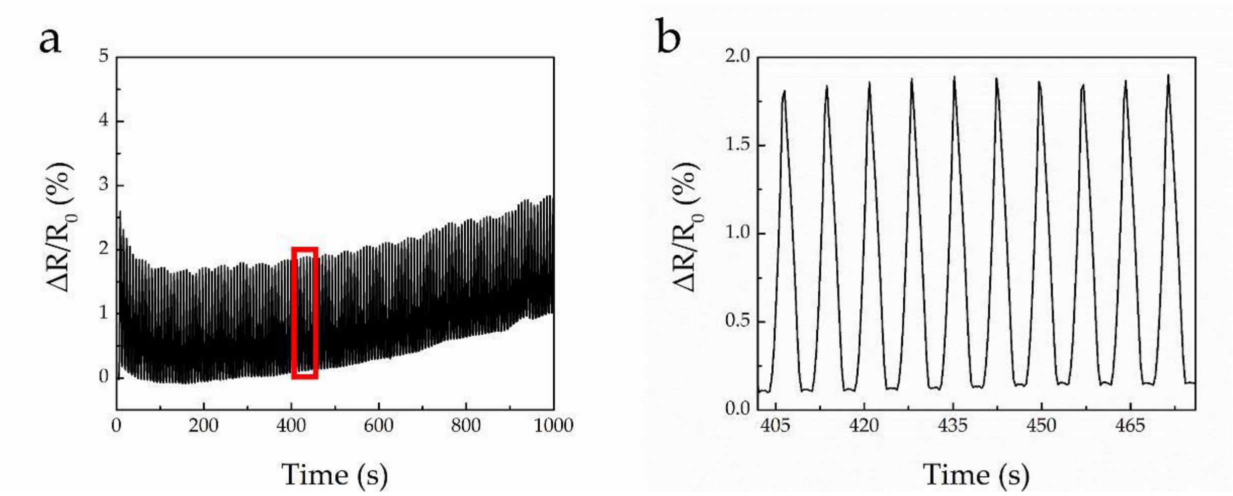

Figure S3. (**a**) The stretching-releasing tests of the strain sensor; (**b**) The stretching-releasing tests of 10 cycles.

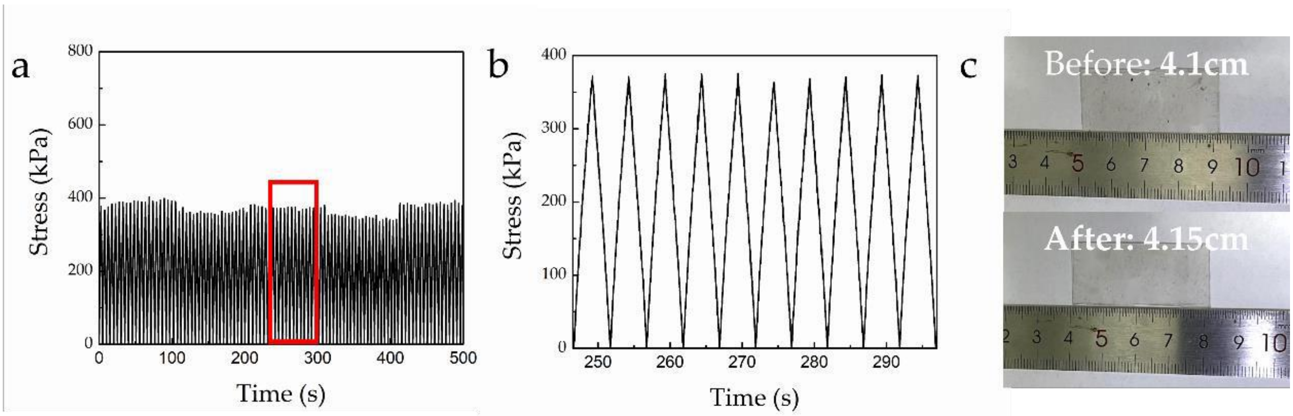

Figure S4. (**a**) The stress variation of the AgNWs/PDMS film in a typical bending cycle of 0-30% strain; (**b**) The stress variation for 10 cycles; (**c**) Photographs of the stretching process.

Table S1. Recovery rate of recently reported PDMS strain sensors.

Sample	Recovery (%)	Strain (%)	Ref
PDMS/VACNT	1.75	30	1
PDMS/CNF	2.54	50	2
Au/Ti/PDMS	3.7	35	3
AgNWs/PDMS	1.22	30	This work

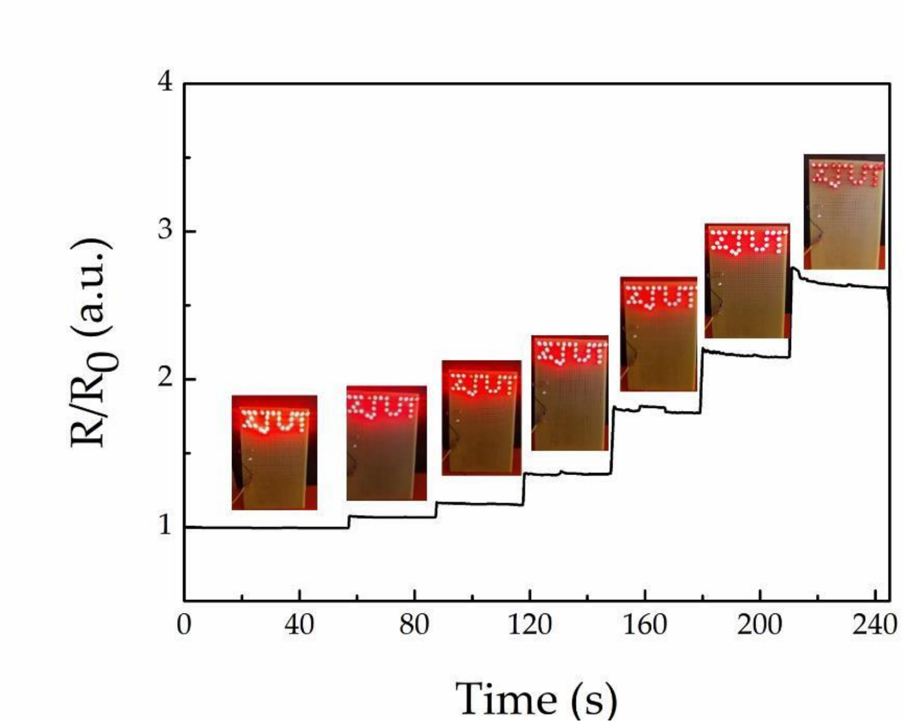

Figure S5. Brightness change of LED lamp during strain sensor stretching at 0%, 2%, 4%, 6%, 8%, 10%, 15% strain.

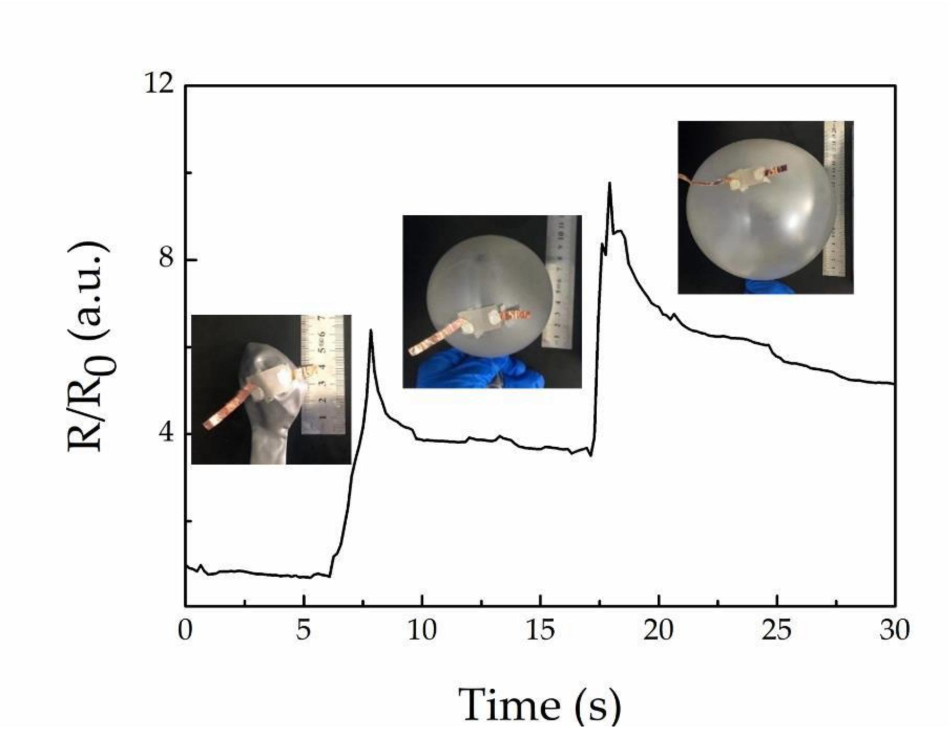

Figure S6. Resistance changes of strain sensor during balloon inflation.
